# Thickness of Polyelectrolyte Layers of Separately Confined Bacteria Alters Key Physiological Parameters on a Single Cell Level

**DOI:** 10.3389/fbioe.2019.00378

**Published:** 2019-12-04

**Authors:** Iaroslav Rybkin, Dmitry Gorin, Gleb Sukhorukov, Aleš Lapanje

**Affiliations:** ^1^Department of Environmental Sciences, Jožef Stefan Institute, Ljubljana, Slovenia; ^2^Remote Controlled Theranostic Systems Lab, Institute of Nanostructures and Biosystems, Saratov State University, Saratov, Russia; ^3^Jožef Stefan International Postgraduate School, Ljubljana, Slovenia; ^4^Department of Reactive Transport, Helmholtz Zentrum Dresden Rossendorf, Institute of Resource Ecology, Leipzig, Germany; ^5^Center for Photonics and Quantum Materials, Skolkovo Institute of Science and Technology, Moscow, Russia; ^6^School of Engineering and Materials Science, Queen Mary University of London, London, United Kingdom

**Keywords:** time-lapse confocal microscopy, polyelectrolytes, layer-by-layer encapsulation, electrostatic interactions, cell surface modification

## Abstract

Confinement of bacterial cells in a matrix or in capsules is an integral part of many biotechnological applications. Here, the well-known layer-by-layer method of deposition of a polyelectrolyte film a few nanometers in thickness to confine separated bacterial cells in permeable and physically durable shells has been examined. Due to the physical properties of such a confinement, we found that this method enables investigation of effects of physical barriers against mass gain and cell division. Using the method of time-lapse confocal microscopy, we observed a prolonged lag phase, dependent on the number of polyelectrolyte layers. In the confinement, both the GFP fluorescent signal from the leaking T7 promoter and the cell size were increased by factors of more than five and two, respectively. This creates a paradigm shift that enables use of mechanical entrapment for control of bacterial cell physiology and opens possibilities of controlling the division rate as well as gene expression. These effects can be attributed to the perturbation of the sensing of the cell size, which results in disproportional synthesis of a cell envelope impinging the intracellular material and compels cells to grow rapidly. In addition, the charged surface of cells enables prolonged intercellular physical interaction and results in spherically shaped microcolonies.

## Introduction

Confinement of bacterial cells in matrices or capsules is an integral part of many applications in biotechnology. The development of different confinement methods of microbial cells is in demand in many industrial and agricultural processes such as microbial based biofertilization in sustainable agriculture, bioaugmentation for water processing or wastewater treatment using carriers in packed, fluid bed reactors or sand filters. In addition, such confinement can be used in medicine to providing protective barriers for probiotic bacteria against harsh conditions in the human gut. Protection of probiotic bacteria is currently gaining much interest due to the most recent discoveries of the importance of healthy human microbiome on human health (Everard and Cani, 2013). Currently, there are many different well-established methods of microencapsulation of microorganisms that are based on extrusion, emulsion, spray drying, electrospinning and other approaches that have been reviewed in the literature (Martín et al., 2015). One of the less conventional methods used for bacterial encapsulation, contrasts with other methods, which enables tailor-made surface modification of bacterial cell, is based on deposition of polyelectrolytes over the surface of the cell using a layer-by-layer (LBL) approach (Hillberg and Tabrizian, 2006). Recently, this approach of electrostatic modification of the bacterial surface has resulted in better adherence of probiotic bacteria to the surface of gut epithelia (Anselmo et al., 2016) and when cells are made in an LBL manner they can enhance the efficiency of vaccination due to the better presentation of the vaccine to immune cells (Speth et al., 2016). The surface modification of single bacterial cells by such a simple method can be a good alternative to the genetic modifications designed to equip cells with different antigen presenting enhancers. When bacterial cells are entrapped within a matrix it has an impact on their physiology, which is mostly attributable to the lower diffusion of nutrients, metabolites, and other charged molecules (Lieleg and Ribbeck, 2011). Using well-established matrix entrapment methods the induction of fermentation activities, the ability to externally induce expression of specific proteins, for example green fluorescent protein (GFP) and the germination of spores have been observed and it was shown that it causes the “skin effect” of encapsulated cells. It is speculated, but not further investigated, that the skin effect is attributable to the increased permeability of the cell lining as a response to the less permeable capsule wall. It has also been shown that confinement induces quorum sensing genes to reach characteristic cell densities sometimes even at the single cell level (Carnes et al., 2010).

LBL entrapment of bacterial cells is however different from entrapment in a matrix such as hydrogel (e.g., alginate, chitosan). LBL entrapment represents the entrapment of a single cell within a strong mesh of thickness in the nanometer range with pores that enable nutrients to pass freely but which keeps cells mechanically separated from the surrounding microenvironment. The polyelectrolytes (PE) deposited on the bacterial cell surface by the LBL method result in tailor-made layered capsules in which each of the layers is only 2–3 nm thick (Hillberg and Tabrizian, 2006; Franz et al., 2010; Fakhrullin and Lvov, 2012). The number of layers determines the thickness and strength of the final capsule (Kolasinska et al., 2007). The capsules formed in this way can tolerate a force of more than 300 MPa (43,500 psi) (Gao et al., 2001; Vinogradova, 2004; Fery and Weinkamer, 2007), and the layers are porous, enabling approximately 4–10 kDa large polar molecules to pass unobstructed (Georgieva et al., 2004). Under physiological conditions they are permeable for molecules of up to 75 kDa (Tong et al., 2005). In contrast to the diffusion properties of entrapment matrices based on gels such as alginate, increasing the thickness of the capsule by incorporating more nano-sized layers of PEs (polyelectrolytes) does not significantly affect the diffusion of molecules (Georgieva et al., 2004; Kozlovskaya et al., 2011).

In view of these properties of the LBL layers, our main aim was to determine effects of an LBL shell consisting of different numbers of layers on growth and division, metabolism and colony establishment of separated *Escherichia coli* cells. It is known that cationic polyelectrolytes can be toxic for bacteria (Kügler et al., 2005), and accordingly we used here a strain that does not show toxic response after being exposed to the highly charged polyelectrolytes and enables us to observe the aforementioned physiological parameters resulting from the mechano-physical interactions of polyelectrolytes with bacterial cells. We also adapted an LBL procedure since in most cases when bacterial cells are used as an LBL template, formation of aggregates usually results (Hillberg and Tabrizian, 2006; Franz et al., 2010; Fakhrullin and Lvov, 2012), and precludes observation of single cells. Accordingly, our specific aims were to (i) prepare a method for time-lapse observation of the physiology of single cells covered with polyelectrolytes, (ii) determine the effects of physical constraint on growth, division and constitutive expression, and (iii) assess the effects of the LBL shell on the process of the microcolony formation.

## Materials and Methods

### Bacterial Strains and Growth Conditions

In all experiments we used non-motile cells of Escherichia coli top 10 strain [F– mcrA Δ(mrr-hsdRMS-mcrBC) Φ80lacZΔM15 ΔlacX74 recA1 araD139 Δ(ara leu) 7697 galU galK rpsL (StrR) endA1 nupG], transformed with pRSET-emGFP plasmid (Thermo Fisher Scientific Corp.) and standard electroporation procedures (Sambrook et al., 1989). The plasmid contains T7 promoter regions upstream of the emGFP reporter gene and ApR cassette. Since the cells are deficient in T7 polymerase, the GFP is transcribed only on a basis of the leakage of the promoter leakage. The transformants were cultivated at 37°C on nutrient agar (NA) plates (Sigma-Aldrich) supplemented with ampicillin (100 μg/ml, Sigma-Aldrich)—NAamp.

Prior to the experiments, we prepared overnight liquid cultures from a single colony in NBamp medium. One milliliter of this culture was transferred into 100 mL of the fresh medium and incubated until optical densities appropriate for conducting the particular experiments were obtained. All liquid cultures were incubated with shaking at 37°C and 150 rpm.

### Determination of the Appropriate Growth Phase of Bacterial Cells for PE Deposition

For efficient polyelectrolyte deposition, the electrostatic properties of the surface of bacterial cells in different growth stages were determined. The charge densities (ZN) and the electrostatic softness parameter (1/λ) of bacterial cells were determined from a non-linear regression analysis of the ionic strength-dependent electrophoretic mobilities (ISDEM) of bacterial cells using Ohshima's soft particle equation as a model (Ohshima, 1995).

To obtain the ISDEM, the cultures were washed 3 times in 0.00062 M NaCl solution. Then 100 μl of washed culture was mixed with 900 μl of the 0.00062 M NaCl solution. The electrophoretic mobilities of bacterial cells were measured using an ELS device (Zetasizer Nano, Malvern, USA). The ionic strength of the suspension of bacterial cells within the measurement cuvette was automatically altered by titration with an MPT-2 titrator. Measurements were made within the linear gradient of ionic strengths of NaCl from 0.00062 to 0.11 M in 12 steps of 0.0091 M per step by addition of a 0.155 M solution of NaCl. The data was obtained from 3 experimental replicates in which each of the measurement was acquired from the accumulated values of 70 separated ELS values. In all ELS measurements the polarities of electrodes were fixed, with an automatically adjustable voltage. The approximate ionic strengths were obtained from the titrator and the exact values for the cell suspensions within the cuvettes and were determined on a basis of conductivity values obtained in each ELS measurement. The ionic strengths in the ELS measurements were calculated on the basis of the standard curve values obtained from the measurement of the conductivities of different concentrations of NaCl solutions in water.

### Preparation of PEs for Layer-by-Layer Encapsulation

We used the negatively charged sodium poly(styrene sulfonate) (PSS) with MW = 70,000 and poly(ethyleneimine) PEs (PEI), with MW = 750,000, both from Sigma-Aldrich, to encapsulate bacteria based on electrostatic principles (Sukhorukov et al., 1999). The solutions of PEs in Milli-Q (ultrapure water type 1) water (2.5 mg/ml, pH 7 adjusted by NaOH or HCl) were prepared by solubilizing PEs, initially by stirring and then by the sonication (35 kHz, 100 W) for 15 min. In experiments using labeled PEI with tetramethylrhodamine isothiocyanate (TRITC) the PEI was labeled using NHS ester labeling of amino biomolecules (Lomant and Fairbanks, 1976). Briefly, the TRITC (11 mg in 11 mL DMSO solution) was added to a PEI solution (2.5 mg/ml in 20 mL of water) in a 50 mL tube and incubated for 4 h at room temperature with constant stirring. To remove the residual dye, the solution after labeling, was dialyzed for 3 days using a dialysis tube (Orange Scientific) with nominal molecular weight limits between 12 and 14 kDa.

### Encapsulation Procedure

Before the procedure, cells were grown at 37°C by shaking at 150 rpm until OD_660_ reached 0.2. Cells were concentrated by centrifugation of 50 mL of the culture at 5,000 g for 6 min. To wash out residues from the medium the pellet was washed three times by resuspension in 30 mL of 0.9% NaCl solution and centrifugation of the suspension at 3,000 g for 3 min.

To prevent formation of aggregates of the cells, we determined the parameters associated with efficient single cell PE deposition. First, we deposited PEI on cells by adding a 0.25% solution of PEI at pH 7 (adjusted with HCl) in Milli-Q water to the washed cells (OD_660_ 1.2) in a 1:1 v/v ratio. This suspension was incubated at room temperature for 5 min. Unattached PEI was removed from the suspension by centrifugation at 900 g for 2 min. The obtained pellet was washed twice by gentle addition of 1 ml of 0.9% NaCl over the pellet taking care to avoid dissolving it. After washing, the PEI covered cells were resuspended in 0.9% NaCl solution. We then added a PSS layer to the PEI covered suspension using a solution in Milli-Q water of PSS at pH 7 (adjusted with NaOH) in a 1:1 v/v ratio. The washing step was the same as was used with PEI, except that 3 min centrifugation at 1,500 g was used to obtain a sufficiently firm pellet to permit washing by pipetting. By repetition of these two steps, we were able to deposit up to 4 such bilayers on the surface of bacterial cells while keeping their aggregation low. The aggregation was monitored by microscopy.

### Effect of Encapsulation on Growth of Cells and GFP Fluorescence Signal

To measure the growth of the population of bacterial cells we inoculated 5 μl of the (OD_600_ = 0.38) uncovered, the control population, or PE covered with the 1, 2 or 3 bilayers, into the microplate wells containing 200 μl of fresh and sterile NBamp media. During the 16 h incubation period at 37°C with vigorous discontinuous shaking prior to each measurement, we measured the OD_600_ every 20 min and obtained the fluorescence signal at 485/528 nm. The fluorescence signals and optical densities obtained in this way were normalized to the fluorescence signal from the control population and the OD_600_ of the media, respectively. Each experiment was performed in triplicate and Student's *t*-test was used for statistical evaluation.

### Time-Lapse Confocal Microscopy (TLCM)

To enable precise measurement by TLCM, it was necessary to distribute cells on a thin planar surface and provide them with appropriate growth conditions to support a few hours of observations. Accordingly, cells were distributed on an NA solid medium within a simple chamber prepared according to a published method (de Jong et al., 2011), with modifications. Briefly, each chamber was created on a surface of a clean cover slip (76 mm × 26 mm) attaching one surface of a rectangular frame made from the double-sided tape 5 cm long × 2 mm wide by cutting out a rectangular shaped 1 cm × 3 cm aperture. The sterile NA was solvated by heating in a microwave oven, then rapidly distributed inside the frame by pipetting. Before the NA gelled, the slide glass was put on top of the medium touching the frame. This formed a smooth and even NA surface confined between two glass surfaces and separated by the thickness of the tape. We left this provisional chamber in a refrigerator for 10 min at 4°C to form a solid NA matrix. The non-attached slide glass was then removed, the protective layer of the double-sided tape was stripped off and then 4 μl of culture, with a final OD_660_ of ~0.4 was evenly distributed over the exposed NA surface. Finally, the chamber was sealed with by attaching the cover slip to the sticky surface of the frame enclosing the NA.

The chamber with the cells was equilibrated at 37°C for 15 min and then transferred to a pre-warmed confocal microscope (a Leica TCS SP8X confocal laser scanning microscope equipped with temperature control system cube and a box thermostated at 37°C). The TLCM was performed at 1000x magnification using an objective lens (HCX PL APO 100x/1.44 OIL) immersed in oil. We observed cell growth and division using the excitation at 525/36 and emissions 525/41 and 605/60 with 850–900 and 750–800 V gains for emGFP and TRITC fluorescence, respectively. The morphology of cells and the quantity of cells were observed using white light and a condenser as the objective lens.

### Analysis of TLCM Micrographs

To determine the growth properties of the cells entrapped in the PE layers, we analyzed the pictures using Fiji software (Schindelin et al., 2012). Prior to the experiment, we selected fields occupied by similar amounts of cells under the microscope. From the fluorescent images that were obtained forming a hyperstack we discarded those that were of low contrast or unfocused. The prepared hyperstacks of the images were then converted to a stack of binary images using the “make binary” plugin. On such prepared images we measured the surface area of cells at consecutive time points using the “analyze particles” plugin to determine temporal changes of the cell biomass. The experiments were performed in triplicate and *t*-test was used for statistical comparison of amount of cell biomass per time of each group of cells with different numbers of deposited PE layers. Based on the data that was obtained, we plotted the growth curves and analyzed them with the R package, Growthcurver (Sprouffske and Wagner, 2016). We calculated growth curve properties such as the growth rate (r), the maximal population size (K), the time (tmid) needed to reach K/2, the time at the point of the fastest division rate (tgen) and the area under the curve (AUC) parameters. The λlag parameter was obtained from the intersection point calculated from linear equations of two regression lines from data points of exponential and lag phases, respectively. The last data point belonging to the set of points within each of these two phases was determined by calculation of the regression coefficient of lines fitted to the data points (R). The lag phase points were determined by starting linear regression calculations from the first point on the graph and using all points until reaching the data point that significantly decreased the R value. In the linear regression calculations of the exponential phase we used the point that was not included in the lag phase as the starting point and all the points up to the last point that did not significantly decrease R.

## Results

### Effects of the Entrapment on the Cell Growth and Division

**Figure 1:**
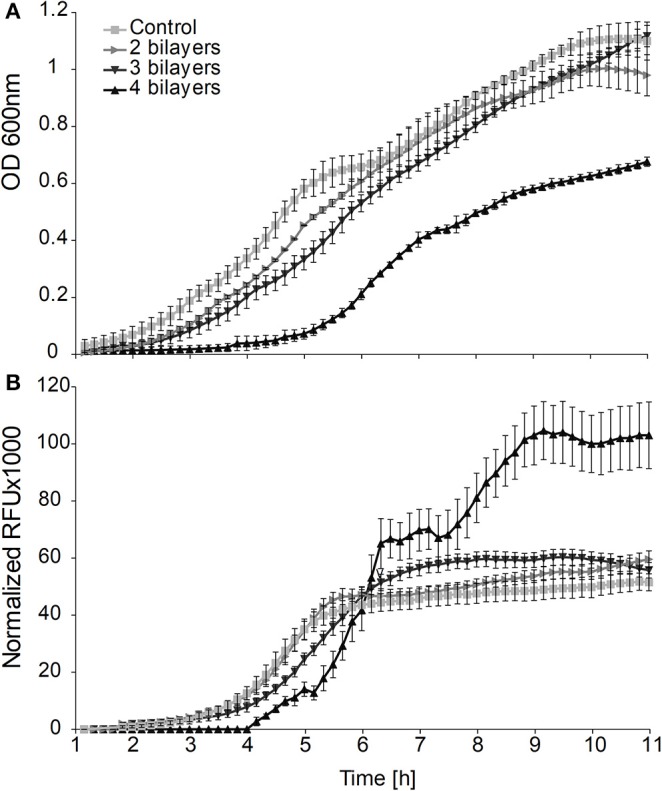
**(A)** Effects of the entrapment of cells in layers of polyelectrolytes on growth and **(B)** GFP fluorescence produced from the promoter leakage without induction of the bulk population of bacterial cells.

By analysis of the growth curves on the population level we observed a significantly prolonged lag phase (Figure 1A) for cells on which we deposited more than one PE layer. The longest delay of the lag phase (λ = 5 h) was observed when 4 bilayers had been deposited. Although the durations of the lag phase of the populations of cells covered with 2 or 3 bilayers were not significantly different from each other, they both showed significantly longer lag phases than the control population of untreated cells (control λ = 1.5 h, 2, and 3 bilayers λ = 2.5 h).

The delay of the exponential phase is caused by either (i) the toxicity of PEs that increases the proportion of killed cells within the inoculum of entrapped cells or (ii) the interference of entrapment with the growth and division of live cells. Accordingly, to resolve this ambiguity, we performed growth experiments on the single cell level using a TLCM approach.

**Figure 2:**
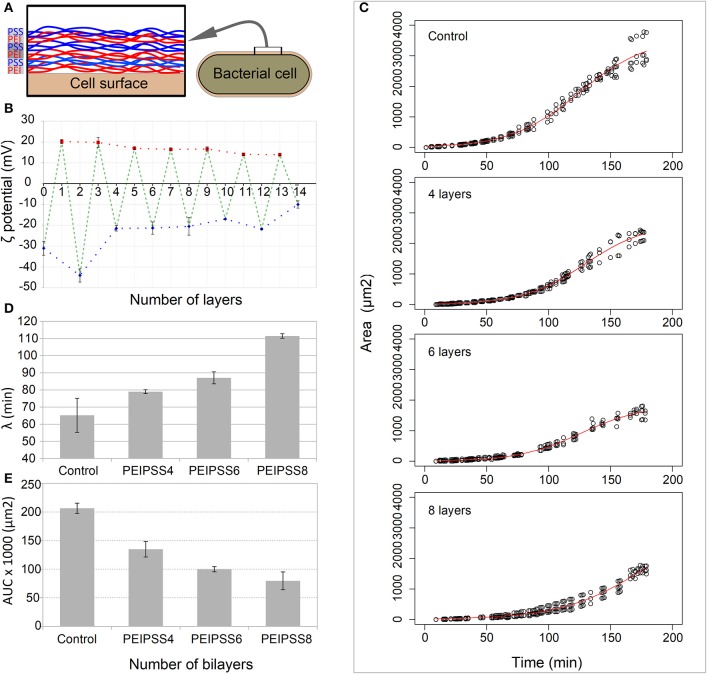
Effects of different number of layers of growth of bacterial cells on a single cell level observed by time-lapse confocal microscopy. **(A)** Bacterial cells were coated with bilayers of polyelectrolytes always ending with a negative one. **(B)** The deposition of layers on bacterial cells was determined by the measurement of the zeta potential. **(C)** The delay of lag phase is correlated with the increasing numbers of layers, and was evaluated by determining the time point of the end of **(D)** the lag phase, the lambda. **(E)** The decreased area under the curve for more than 4 layers resulted from the curve shift toward the right.

Since covering of the cells with PEs tends to form aggregates (see Supplementary Material Data Sheet 1), we optimized the conventional method to avoid aggregation that can interfere with the diffusion of nutrients. Precise coverage using the updated method resulted in a switch of the surface charge of the bacterial cells from the initial −30 mV to the 20 mV. Using this approach, we were able to avoid aggregation (see Supplementary Figure 1) when we deposited as many as 10 layers of PEs (Figures 2A,B).

Using TLCM on such covered cells, we observed growth in more than 99% of cells within each of the fields of view in both the control and entrapped population of cells and we failed to observe any toxicity attributable to the LBL entrapment. However, we observed a prolongation of the lag phase which was proportional to the number of the PE layers (Figure 2C). The longest delay of the lag phase was observed for cells entrapped in 8 layers (λ = 111.5 ± 1.3 min), whose lag phase is almost 2 times longer than that in the control population of cells (λ = 65.2 ± 9.9 min) (Figure 2D). The acceleration of the growth, which is the transition from the lag to the exponential phases, is overall lower for cells covered with PEs (*p* < 0.1) and decreases proportionally with the increasing number of added layers (see Supplementary Table S1). Other growth curve properties such as the tmid and AUC parameters (Figure 2E) of the populations of cells covered with the PEs were significantly smaller than in the control population of cells. On the other hand, the maximum growth rate (r) and generation times were not significantly different in all experiments except those in which the cells were covered with 8 layers of PEs where we observed slower growth rate and consequently longer generation times (Supplementary Table S1, *p* < 0.05).

### Escape of the Cells From the LBL Layers

**Figure 3:**
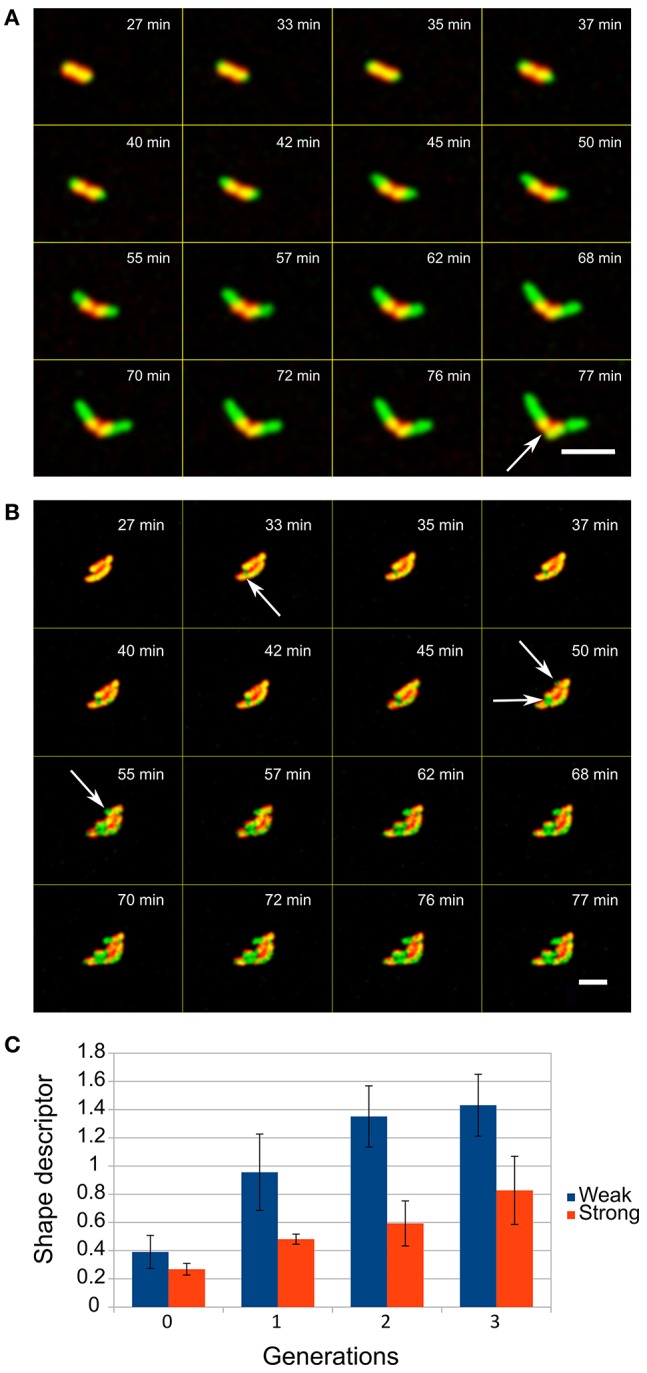
Escape of *E. coli* cells from the LBL layers when entrapped in **(A)** low and **(B)** high number of layers (red is LBL shell, green is GFP producing cells, overlap regions are yellowish), scale bar = 5 μm. The entrapment of cells in different numbers of layers (up to 6 layers is weak and a shell with 8 layers is a strong shell) determines how compact microcolonies can be formed. **(C)** The shape descriptor is an estimate parameter of circularity where zero values represent an optimal round shape (see Methods). Two-tailed *t*-test of encapsulated and control cells comparison showed significant difference (*P* < 0.05) in the shape descriptor from the first generation onward.

We measured the number of layers determining the escape of the cells from the capsules by analyzing the growth of each of the covered cells. With up to 6 layers, cells escape by their elongation which initiates cracking of the capsule at the distal positions above the cell tips leaving a belt of PE layers surrounding the cell (Figure 3A). However, when more layers were deposited on bacterial cells this resulted in the escape of the cells by decreasing the overall integrity of the capsules, not only on distal part, which was observed as occurrence of several green fluorescent patches of the cell material within the yellow-reddish walls of the capsules (Figure 3B). Then small bulges of the cell material were formed, from which cells began to grow outward from the capsules.

Analysis of the events after the initial escape of cells showed that a deposit of 8 layers of PEs on cells results in formation of significantly more spherical microcolonies with smoother edges than in populations of cells where 6 or fewer layers had been deposited (*t*-test, *p* < 0.05, Figure 3C).

### Effects of Entrapment on the Shape of Bacterial Cells and GFP Fluorescence

On the population level we obtained the maximum intensity of the fluorescent signal in the middle of the exponential phase from all of the cultures with either entrapped or free cells. However, the culture with cells entrapped in 3 and 4 bilayers showed 1.2 and 2.1 times higher fluorescence signals, respectively, than the signals obtained from suspensions of untreated cells (Figure 1B).

**Figure 4:**
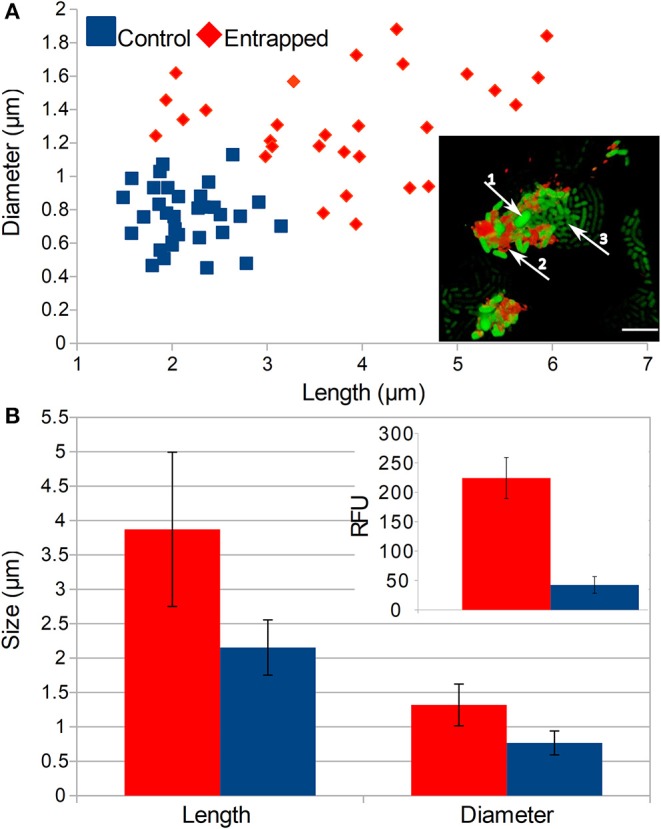
Effects of LBL entrapment on the morphology of cells. **(A)** Comparison of populations of normal and entrapped cells and the size and position of entrapped cells is in the inset picture (1—enlarged cells, 2—TRITC stained polyelectrolytes, 3—normal cells that escaped from the entrapment, scale bar = 5 μm). **(B)** Comparison of the average length and diameter of entrapped and control cells and intensities of the fluorescent signal from GFP (see inset picture). Populations of entrapped and normal cells are represented with red and blue colors, respectively. Two-tailed *t*-test of encapsulated and control cells comparison showed significant difference (*P* < 0.05) in RFU as well as cell size.

On the single cell level, we observed that numerous cells entrapped inside the more than 6 PE layers were aggregated and failed to divide (Figure 4A, insert). These cells formed a distinctive population of cells that were either (i) of the same length as the control but with larger diameters (ii) longer than the control, but of the same diameter or (iii) bigger in both dimensions than the normal *E. coli* cells (Figures 4A,B). On average, both the size and diameter of entrapped cells were significantly larger than those of normal cells (*p* < 0.01). Those larger non-dividing cells also showed 5 times higher intensity of the fluorescent signal than normal *E. coli* cells that were not entrapped (*p* < 0.01).

## Discussion

The cell acts as a micrometer sized particle with a persistent negative electrostatic charge. We found this property to be of high biotechnological value since different oppositely charged molecules can be deposited on the cell surface, enabling simple and robust surface modification of the cell. The modified cellular surface can be positively charged due to the polyelectrolyte deposition, and this can enable fast attachment of cells on the surfaces, resulting in an immobilized biomass. The control of division by polyelectrolyte capsules can determine the exact and synchronous start of the biotechnological process. Mechanical forces opposing the division process increase the expression that can result in higher yields. By mixing oppositely charged cells the aggregates can be formed and perhaps can enable more efficient growth of co-cultures due to the spatial distribution of the different cells. For the most efficient encapsulation it should be considered that the polyelectrolytes and the bacterial cells must be carefully chosen to prevent aggregations in any particular encapsulation experiment.

In our experiments it was observed that physical constraints cause delay in division and excessive gain of cell size as well as an increased transcription rate of the constitutively expressed operon. Since the confinement modifies the gene expression without chemical induction, it offers new opportunities to control bacterial cell physiology, which has not been possible previously. On the one hand this paradigm shift opens possibilities of controlling the division rate as well as gene expression with mechanical constraints, and on the other hand, since cells become electrostatically adhesive, surface modification can be used for spatial positioning of individual cells on surfaces or in aggregates involving either single species or complex consortia (e.g., surface attached biomass or artificially formed flocs).

The observed delay in the onset of the division of cells (see Figure 2) until disruption of the PE capsule and their escape from the entrapment by the cell division (see Figure 1) is caused only by either decreased diffusion flux of nutrients or increased mechanical strength of the PE shell, since the third possibility, toxicity of the PE layers, was not observed. The deposited polyelectrolyte film can affect the physiology of the cell due to the (i) diffusion, resulting from thickness of the film and its pore sizes, and (ii) strength, which is dependent on the charge densities of the polyelectrolytes, the number of their layers and branching as well as ionic strength of the media. The diffusion property of LBL walls is less likely to cause delayed growth, since it has been shown in several studies of activities of LBL encapsulated enzymes that enzymatic substrates larger that 6 kDa (in some cases up to 75 kDa), such as short peptides and dextran molecules, can freely pass the PE wall (Antipov et al., 2002; Qiao et al., 2005). Since we used NA, more than 70% of which consists of peptides with mass below 1 kDa, the PE layers cannot affect the flux of the nutrients. Moreover, the limiting diffusion flux for growth of *E. coli* cells should be two orders of magnitude lower than the lowest reported diffusion rate for LBL layers (D = 10^−12^ cm^2^s^−1^ = 10^−4^ μm^2^s^−1^, t ≈ 80 ms of small molecules of 332 Da) (Kozlovskaya et al., 2011) since only ~10^9^ glucose molecules are transported in 2,400 s during the most active growth (Milo et al., 2009) and the PE layers are thin (1 bilayer is 4 nm) (Sukhorukov et al., 1998; Kolasinska et al., 2007). Therefore, during the most intensive growth, up to 8 PE layers (16 nm thick) can adequately provide the requisite amount of the necessary glucose molecules. In addition, in our experiments, since cells were in a lag phase, a stage of slow growth, they needed a lower flux of nutrients, and the NA medium #2 is composed mostly of short proteins and amino acids that are transported and metabolized more slowly than glucose. These assumptions are also consistent with our observations indicating that growth of cells covered with a single bilayer was not significantly different from that of the control.

Although each separate bilayer in the 8 bilayer structure does not obviously contribute to the decrease of the diffusion properties (Kozlovskaya et al., 2011), the strength of the capsule is significantly increased by 0.05 μN per bilayer (Vinogradova, 2004). In LBL capsules composed of PEs similar to those used in our study, a Young modulus of between 170 and 300 MPa was determined (Gao et al., 2001; Vinogradova, 2004; Fery and Weinkamer, 2007). Hence, it is intuitive that the forces which counteract the cell growth result in a prolonged lag phase, although the LBL cores in the aforementioned studies are not cells and the absolute values cannot be directly applied to our system. However, we do observe the growth delay and number of layers are therefore significantly correlated, probably due to the progressive increase of the strength of the capsule with the number of layers (see Figure 2). In addition, the number of layers also determines the type of the escape. Cells can escape from the distal parts of capsules when they are enclosed in a few layers but if more than 4 bilayers are added, they will tear the capsule in the middle. Although the tips of the *E. coli* cells are expected to be more negatively charged due to the increased levels of cardiolipins, which contribute to stronger interaction with the positively charged PEI, the curvature of the capsule is larger at the tips than in the remainder of the cell surface and this causes relaxation of layers due to the Ostwald ripening. Since *E. coli* cells grow from the central region, the surface of the cell must slide along the capsule wall to break the capsule at the tip. This sliding might be possible on account of the lower negative charge densities along the middle part of the cell, since the membrane in these regions contains higher amounts of less negatively charged phosphatidylethanolamine phospholipids (Matsumoto et al., 2006).

When a strong, thick layer is deposited on the surface of the cell, it appears that the adaptive properties of the shape of the *E. coli* cells help the cells to escape from the capsule whose wall is partially perforated by the tearing. The squeezing properties of the *E. coli* cells reported by Männik et al. (2009), are crucial for the escape of the cell through the 300 nm narrow pores within the capsule wall (see Figure 3B at 50 min). However, this feature alone would be insufficient for a successful escape, since the initial pores can occur randomly anywhere along the capsule wall and the flexibility and rigidity of the cell should be also considered (Amir et al., 2014). For example, we observed that the partial escape from the pores bends cells in one direction (see Figure 3B at 55 min). The cell then preserves its shape by its elastic response and the remodulation or regrowth of the cell wall, which causes increased forces on the occupied pore on the opposite side of the cell. This pushes the cell through the pore, and also increases the widening of the pore as it enables insertion of the opposite tip of the cell into the pores, which are formed during the consecutive events in the continuous expansion of the cell volume and tearing apart of the capsule wall.

Since different sorts of interferences with the physical intercellular interaction result in the formation of diverse colony shapes (Ishii et al., 2004; Seminara et al., 2012), we showed that with polyelectrolytes we can also interfere with the formation of microcolonies. Here the capsule wall is not uniformly strong (see Figures 3A,B) and after its disintegration, patches of the capsule wall stay attached to the cell surface. This keeps the cells together due to the increased electrostatic adherence of one cell to another, resulting in formation of compact microcolonies (see Figure 3C).

When we added more than 6 PE layers, we made capsules that were strong and not ruptured by the growth of cells. In such strong capsules we observed increases in both the size of cells and GFP expression from the T7 promoter without its induction. Since the cells were not deprived of nutrient, but were constrained, we expected a paradox in which the mechanical constraint prevents cells from growing but at the same time induces their growth. This paradox can occur because bacterial cells sense membrane capacity which can support the normal activity of the cell with the amount of available free membrane lipids in the cytosol. The presence of membrane lipids inhibits ppGpp activity, which results in increased central metabolism involved in the synthesis of cellular components (Vadia et al., 2017). Hence, in our system it is expected that the amount of the free membrane lipids is in excess in the cytosol and this results in constantly engaged anabolic metabolism, forcing cells to synthesize cellular components continuously. This can indirectly increase GFP synthesis (see Figure 4) through the increased number of (i) plasmids and (ii) RNA polymerases, resulting in the decreased specificity of the RNA polymerases (RNAP) transcription.

The process of cell division and protein dilution rate as well as gene copy numbers are usually disproportionate in one cell, since chromosomes can be synthesized faster than cell division can occur (Klumpp et al., 2009). Consequently, in rapidly dividing cells, which contain the most disproportionate levels of cellular components, it was observed that the level of constitutive expression is higher due to the disproportionate number of chromosomes in one cell. In our GFP expression system, the T7 regulated operon leaks without any regulation, and its transcription can be dependent only on the cell status, the operon therefore acting as a constitutive promoter. Moreover, when using physical constraint by depositing PE layers, the growth and division of the cell are limited, and one can expect even more chromosomes producing more RNAP and at the same time due to the disproportionation more plasmids per cell can be expected as well. Prevention of separation into daughter cells decreases dilution of the intracellular proteins including RNAP, which can in turn decrease the specificity of transcription and increase leakage, resulting in an increased GFP signal. The amount of RNAP is therefore dependent on the growth rate. Faster growth rate produces more RNAP, either free, transcribing or non-specifically bound (Klumpp and Hwa, 2008). Since the dilution of proteins by division is prevented and anabolic metabolism is increased as a result of the entrapment, the levels of unbound RNAP are even higher, inducing higher rates of non-specific transcription including the GFP gene in this simple genetic system.

In addition, we demonstrated here for the first time the effects of physical constraints on bacterial physiology on the *E. coli* model organism. Our results open a new vista of the physiological responses of bacteria in different environments such as biofilms, porous materials, infected tissues, and intracellularly inside amoebas or macrophages, where mechanical constraint can play a role.

## Conclusion

Careful deposition of polyelectrolytes enables a single cell encapsulation in a capsule with the wall thickness of a few nanometers. The polyelectrolytes are not toxic to *E. coli*, but due to the high mechanical strength of the capsule wall the physiological properties of the cell such as lag phase, colony formation and leakage expression are affected. Accordingly, measurements at a single cell level showed that the number of polyelectrolyte layers correlates positively with the duration of the lag phase. Over time, cells can escape from the shells at the distal position in weaker capsules and in stronger capsules the cracking process of the polymer wall is randomly distributed. Due to the intracellular electrostatic interactions, the escaped bacteria formed more spherical microcolonies than non-encapsulated bacteria. The mechanical stalling of the division also effects the cellular molecular physiology and biochemistry, since we observed high increase in GFP fluorescent signal from the leaking operon and 2–3 times increased length and width of the encapsulated cells when their division process was stalled. We can conclude that the encapsulation of cells described here can be a versatile instrument for the modification of the cell surface without applying genetic tools and might be suitable when the genetically modified organisms may not be used.

## Data Availability Statement

All data generated and analyzed during this study are included in this article/Supplementary Material. Raw datasets are available from the corresponding author upon reasonable request.

## Author Contributions

IR executed the experimental part and performed statistical analysis as well as contributing partially to the writing. DG and GS were involved in the conceptual design of the experiments and they contributed to the critical reading of the manuscript. AL developed the idea and concept, designed and executed the experiments, and prepared the manuscript.

### Conflict of Interest

The authors declare that the research was conducted in the absence of any commercial or financial relationships that could be construed as a potential conflict of interest.

## Abbreviations

ApR: Ampicillin Resistance
auc: area under the curve
ELS: Electrophoretic Light Scattering
GFP: Green Fluorescent Protein
ISDEM: Ionic Strength-Dependent Electrophoretic Mobilities
LBL: Layer-by-Layer
NA: Nutrient Agar
NB: Nutrient Broth
OD: Optical Density
PE: polyelectrolytes
PEI: Poly(Ethyleneimine)
ppGpp: Guanosine pentaphosphate
PSS: Poly(Styrene Sulfonate)
RNAP: RNA polymerases
TLCM: Time-lapse confocal microscopy
TRITC: Tetramethylrhodamine isothiocyanate
ZN: Charge densities.
