# Supplementary material for: Thickness of Polyelectrolyte Layers of Separately Confined Bacteria Alters Key Physiological Parameters on a Single Cell Level
Source: Front Bioeng Biotechnol. 2019 Dec 4;7:378. doi: 10.3389/fbioe.2019.00378 (PMC6904277; doi:10.3389/fbioe.2019.00378)
Supplement: Supplementary file 1 [file Data_Sheet_1.docx]

Supplementary Material

Thickness of polyelectrolyte layers of separately confined bacteria alters key physiological parameters on a single cell level

# Supplementary results

## Entrapment procedure

Our goal was to observe the effects of mechanical restriction of the PEs while avoiding diffusion restrictions as much as possible and consequently, it was necessary to keep cells separated. The current procedures of deposition of PE on the surface of bacterial cells are prone to the formation of aggregates after separating out the non-attached PEs by centrifugation (Fig. S1). We improved the method by selecting the cells in an appropriate growth stage and modification of the centrifugation step. The cells in the exponential phase showed the highest electrophoretic mobilities (Figure S1A) and significantly more negative surface charge (Figure S1B) than those in the lag phase as determined through Ohshima's soft particle model (Duval and Ohshima, 2006). Although we also tested the LBL procedures in all three phases, according to our expectations we were able to prepare the most dispersed cell suspension when the cells were in the exponential phase.

The most critical step was the repeated washing out of the excess PE by centrifugation. This resulted in excessive aggregation of cells and consequently, we developed a protocol in which just one centrifugation was used after PEI deposition on cells and then the supernatant was exchanged 3 times for fresh physiological solution. With this method, cells were efficiently covered with a deposited layer of PE (see changed potential after each deposited layer in Figure 2B). Alternatively, we observed the change of the colour of cells from green to red-orange due to the presence of TRITC stained PEI on the cell surface (Figure 3A). Using this modified approach, we were able to prepare up to 14 layers, or 7 bilayers of PEI/PSS. After more than 7 bilayers were deposited, the bacterial suspension became less stable, which can be observed by steady decrease of zeta potential toward 0 mV with -30mV to -10mV and 20mV to 13mV values for first to the last bilayers, respectively (see Figure 2B). This developed approach resulted in the suspension of LBL covered cells similar to the control (Figures 1S, C and D), which enabled single cell confocal time lapse microscopy experiments.

# Supplementary discussion

According to our preliminary results, we tested different combination of polyelectrolytes and the pair PEI-PSS was chosen since the lowest possible aggregation was achieved. From micrographs of previous reports (Balkundi et al., 2009; Franz et al., 2010; Hillberg and Tabrizian, 2006) and our preliminary tests, it can be deduced that aggregation is a common problem during the deposition of PE on bacterial cells. The removal of the PE by centrifugation and washing out is the most critical step. We suppose the bacterial cells are of lower mass with less momentum than the bigger eukaryotic cells, since the formed clumps of cells can be disintegrated into smaller aggregates containing as few as 10 cells that is equivalent to the size of the average eukaryotic cell. Hence, we decreased the number of centrifugation steps as well as centrifugation force.

The charge density on a surface of bacteria seems to play a role, since we observed that the cells in the exponential phase were easier to encapsulate without aggregation. On one hand this can result from higher charge densities at the same softness parameter than in the lag phase (see Figure S1B) resulting in better PE interaction with the cell surface. On the other hand, it may be due to the lower amount of debris of dead cells and thinner surface layers of extracellular polysaccharides on cells than in the stationary phase. Cells were also needed to be vital whereas in the stationary phase culture cells are deprived of nutrient and the proportion of death cells is increased thus not appropriate for using in our experiments.

However, the larger numbers of layers result in a higher aggregation rate which was observed under the microscope in experiments using more than 8 layers. The results of zeta potential measurements showed the inclination toward zero, which is a sign of a less stable colloidal solution (see Figure 2B). The increased number of aggregates after 8 PE layers can be a result of the more efficient sedimentation of aggregates than separated cells under low sedimentation forces, due to the higher buoyancy and lower charge densities compared to individual cells. Higher charge densities of cells repeal individual cells from surfaces more efficiently than aggregates are repealed, decrease sedimentation and consequently increase the amount of aggregates at each PE deposition step.

# Supplementary Figure


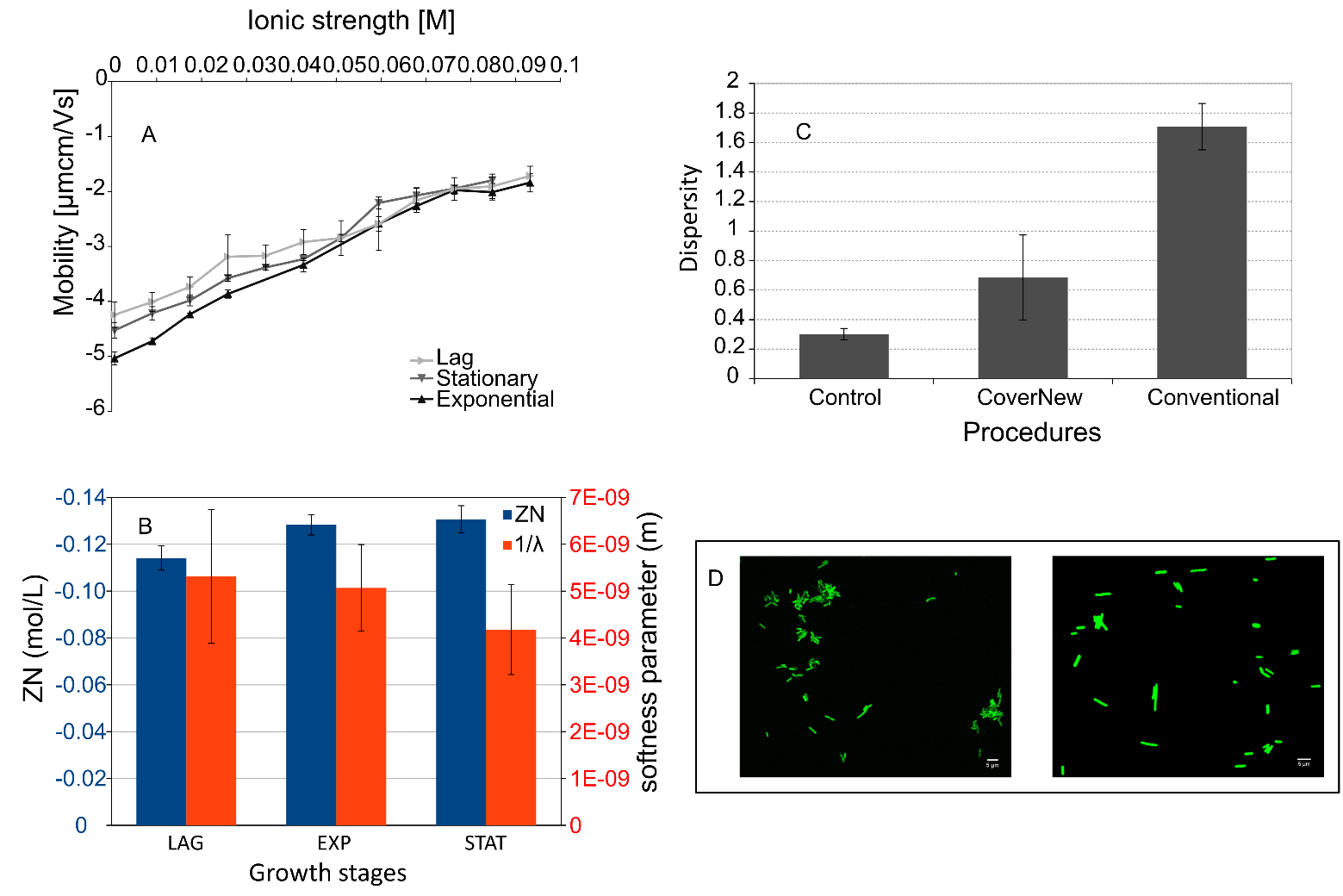


**Supplementary Figure 1.** Improved method of LBL deposition of bacterial cells. (A) Electronegativity of cells within three growth phases measured in NaCl solutions of various ionic strengths, (B) calculated surface charge (ZN) and softness parameters (1/λ) in growth phases (lag, EXP - exponential, STAT - stationary, (C) comparison of the dispersity values of LBL entrapped cells using our improved approach and (D) confocal images of cells entrapped in polyelectrolyte layers using conventional (left) and our improved method (right).

# References

Balkundi, S. S., Veerabadran, N. G., Eby, D. M., Johnson, G. R., and Lvov, Y. M. (2009). Encapsulation of bacterial spores in nanoorganized polyelectrolyte shells. *Langmuir* 25, 14011–14016.

Duval, J. F., and Ohshima, H. (2006). Electrophoresis of diffuse soft particles. *Langmuir* 22, 3533–3546.

Franz, B., Balkundi, S. S., Dahl, C., Lvov, Y. M., and Prange, A. (2010). Layer‐by‐Layer Nano‐Encapsulation of Microbes: Controlled Cell Surface Modification and Investigation of Substrate Uptake in Bacteria. *Macromolecular bioscience* 10, 164–172.

Hillberg, A. L., and Tabrizian, M. (2006). Biorecognition through layer-by-layer polyelectrolyte assembly: In-situ hybridization on living cells. *Biomacromolecules* 7, 2742–2750.
