# Supplementary material for: Thickness of Polyelectrolyte Layers of Separately Confined Bacteria Alters Key Physiological Parameters on a Single Cell Level
Source: Front Bioeng Biotechnol. 2019 Dec 4;7:378. doi: 10.3389/fbioe.2019.00378 (PMC6904277; doi:10.3389/fbioe.2019.00378)
Supplement: Supplementary file 2 [file Table_1.docx]

**Supplementary table S1.** Parameters of the growth curves obtained from time lapse microscopy. N0 - predicted number of cells at the beginning of the experiment, r - maximal growth rate, tmid - time point at which the maximal growth is reached, tgen - generation time at the maximal growth speed, AUC - area under the growth curve, λ - time when culture switch from the lag to the exponential phase, dS(λ)/dt - acceleration of growth at the λ.

| Samples | N0 | r | tmid | tgen | AUC∫S*dt | λ | dS(λ)/dt |
| --- | --- | --- | --- | --- | --- | --- | --- |
| Control | 34.39 | 0.0377 | 114.20 | 18.46 | 206,498.40 | 65.22 | 12.42 |
| PEIPSS4 | 15.32* | 0.0401† | 130.60*,† | 18.54 | 134,904.89*,†,‡ | 79.37†, | 11.40* |
| PEIPSS6 | 11.54* | 0.0404† | 135.19*,† | 17.35 | 99,922.18*,‡ | 87.05*,†, | 10.83* |
| PEIPSS8 | 11.65* | 0.0302* | 181.11* | 22.99* | 79,612.24* | 111.48* | 8.77* |

* significantly different than the control

† significantly different of cells covered with 8 layers of samples from 4 and 6 layers

‡ significantly different between 4 and 6 layers

p<0.05 and grey symbols p<0.1
